# Biweekly oxaliplatin, raltitrexed, 5-fluorouracil and folinic acid combination chemotherapy during preoperative radiation therapy for locally advanced rectal cancer: a phase I–II study

**DOI:** 10.1038/sj.bjc.6603195

**Published:** 2006-05-30

**Authors:** A Avallone, P Delrio, C Guida, F Tatangelo, A Petrillo, P Marone, L G Cascini, B Morrica, S Lastoria, V Parisi, A Budillon, P Comella

**Affiliations:** 1 Department of Medical Oncology, National Tumour Institute, Via M Semmola, Naples 80131, Italy; 2 Department of Surgical Oncology, National Tumour Institute, Via M Semmola, Naples 80131, Italy; 3 Department of Diagnostic Imaging and Radiotherapy, National Tumour Institute, Via M Semmola, Naples 80131, Italy; 4 Department of Pathology, National Tumour Institute, Via M Semmola, Naples 80131, Italy; 5 Department of Experimental of Oncology, National Tumour Institute, Via M Semmola, Naples 80131, Italy

**Keywords:** 5-fluorouracil, oxaliplatin, preoperative radiotherapy, raltitrexed, rectal cancer, tumour regression grade (TRG)

## Abstract

Oxaliplatin (OXA), raltitrexed (RTX), 5-fluorouracil (FU) and folinic acid (FA) have shown activity in metastatic colorectal cancer, radioenhancing effect and synergism when combined. We evaluated a chemotherapy (CT) combination of OXA, RTX and FU/FA during preoperative radiotherapy (RT) in locally advanced rectal cancer (LARC) patients. Fifty-one patients with LARC at high risk of recurrence (T4, N+ or T3N0 ⩽5 cm from anal verge and/or circumferential resection margin ⩽5 mm) received three biweekly courses of CT during pelvic RT (45 Gy). Surgery was planned 8 weeks after CT-RT. Recommended doses (RDs) determined during phase I were utilised in the subsequent phase II trial, where the rate of tumour regression grade (TRG) 1 or 2 was the main end point. No toxic deaths occurred, and severe toxicity was easily managed. In phase II, RDs delivered in 31 patients were OXA 100 mg m^−2^ and RTX 2.5 mg m^−2^ on day 1, and FU 900 mg m^−2^ and LFA 250 mg m^−2^ on day 2. Main severe toxicities by patients were grade 4 neutropenia (23%) and grade 3 diarrhoea (19%). In 71% (95% confidence limits, 52–86%) of patients, TRG1 (13) or TRG2 (9) was obtained. All patients are alive and recurrence-free after a median follow-up of 29 months. Combination of OXA, RTX and FU/FA with pelvic RT has an acceptable toxicity and a high clinical activity in LARC and should be studied further in patients at high risk of recurrence.

Total mesorectal excision (TME) has markedly improved the local control in patients with locally advanced rectal cancer (LARC) (MacFarlane *et al*, 1993). Moreover, the Dutch trial demonstrated that the addition of preoperative radiation therapy (RT) to TME reduced the rate of local recurrence. Nevertheless, the overall survival (OS) was not improved because RT failed to prevent distant metastases (Kapiteijn *et al*, 2001). Furthermore, preoperative delivery of 5-fluouracil (FU) during RT has been proven to further reduce the local recurrence and toxicity compared to postoperative approach, but it did not increase recurrence-free and OS (Sauer *et al*, 2004).

Raltitrexed (RTX), a direct and specific TS inhibitor (Jackman *et al*, 1991), has shown activity in advanced colorectal cancer (Cunningham, 1998). Moreover, RTX has demonstrated radiosensitising properties in preclinical studies (Teicher *et al*, 1998) as well as activity when combined with preoperative or postoperative RT in LARC (Botwood *et al*, 2000; Gambacorta *et al*, 2004b). Interestingly, *in vitro* studies have shown a synergistic activity when RTX is followed 24 h later by FU (Longo *et al*, 1998; Caponigro *et al*, 2001), and a positive pharmacokinetic interaction between RTX and FU has been demonstrated (Schwartz *et al*, 2004). When folinic acid (FA) is added to FU in the combination, an even greater synergism has been observed (Longo *et al*, 1998). Moreover, preclinical studies have shown that the administration of FA 24 h after RTX may reduce its bone marrow and gastrointestinal toxicity (Farrugia *et al*, 2000), which have been the main life-threatening toxicities of RTX in a large multicentre randomised trial (Maughan *et al*, 2002). As a matter of fact, we have treated 53 patients with metastatic colorectal cancer with a biweekly administration of RTX on day 1, followed by FA-modulated FU on day 2, reporting no toxic deaths; severe neutropenia and diarrhoea were absolutely manageable, and a 24% response rate was obtained (Comella *et al*, 2000).

Oxaliplatin (OXA) is a platinum derivative that has shown radiosensitising properties (Cividalli *et al*, 2002), additivity with RTX and synergism with FU (Raymond *et al*, 2002). Clinical studies have shown high response rates for the combination of OXA with either FU/FA or RTX in MCRC (Cascinu *et al*, 2002; Seitz *et al*, 2002; Goldberg *et al*, 2004; Comella *et al*, 2005). Furthermore, the addition of OXA to a biweekly regimen of RTX and FU/FA proved manageable and active in heavily pretreated patients (Comella *et al*, 2002). Recently, several investigators have reported encouraging results with the addition of OXA to fluoropyrimidines during pelvic preoperative radiotherapy in LARC (Gerard *et al*, 2003; Rodel *et al*, 2003; Gambacorta *et al*, 2004a; Aschele *et al*, 2005; Sebag-Montefiore *et al*, 2005). For all these reasons, the combination of OXA, RTX and FU/FA with pelvic RT represents an attractive perspective in the preoperative setting of LARC. In fact, besides the radio-enhancing effect, this regimen takes advantage of the synergism demonstrated between the three agents. Therefore, we performed a phase I–II trial on the biweekly combination of OXA, RTX and FU/FA during RT in patients with LARC.

## PATIENTS AND METHODS

### Eligibility criteria

Eligible patients had a histologically proven previously untreated adenocarcinoma of the extra-peritoneal rectum. In the phase I study, we enrolled patients in clinical stage II or III, or requiring an abdomino-perineal resection (APR). In the phase II study, we accrued only patients at high risk of recurrence: T4, node positive or T3N0 of the lower third of the rectum and/or with circumferential resection margin (CRM) ⩽5 mm by magnetic resonance imaging (MRI) (Beets-Tan *et al*, 2000). Additional inclusion criteria were Eastern Cooperative Oncology Group performance status ⩽2, age ⩾18 years and adequate baseline bone marrow and organ function. Main exclusion criteria were previous malignant tumour, severe heart disease, uncontrolled infection or metabolic disorders, severe neurologic or inflammatory bowel disease. This study was approved by the Independent Ethical Committee of the National Tumour Institute of Naples and a written informed consent was obtained from all patients before enrolment.

### Work-up

Pretreatment work-up included a complete history and physical examination, a complete blood cell count (BCC) with white blood cell differential, a serum chemistry profile, carcinoembryonic antigen assay (CEA), an electrocardiogram, colorectal endoscopy with a biopsy of the tumour, transrectal ultrasonography (EUS), chest X-ray, pelvic and abdominal computed tomography (CT) and liver and pelvic MRI. Blood cell counts were also obtained 8 and 11 days after each cycle of chemotherapy, whereas serum chemistry was repeated weekly.

### Radiotherapy

Conformal RT was delivered by a three-field box technique, consisting of a posterior–anterior and two lateral fields, using high-energy X photons (6–20 MV) by a Varian CD 2100 linear accelerator, to a total dose of 45 Gy over 5 weeks (1.8 Gy × 5 fractions/week) to the reference point according to International Commission on Radiation Units (ICRU) 50–62. Radiation therapy was interrupted only when a severe toxicity occurred. All patients were treated in prone position, and a bellyboard was utilised to minimise the amount of small bowel in the treatment field. Clinical target volume encompassed the tumour, defined by MRI imaging, plus the total mesorectum, the common, internal iliac and obturatorial lymph nodes. Three-dimensional plan was performed with a dedicated treatment planning system after on-line CT virtual simulation and CT-MRI image fusion. We contoured the small bowel, the femoral heads and the bladder as critical organs on all CT slices of every patient (according to ICRU 50 and 62), and we evaluated the relative dose–volume histogram on the treatment planning console. A quality control was assured by a weekly portal imaging verification of all fields, and adherence to the protocol was checked by portal imaging verification and matching all the fields with digitally reconstructed radiographs.

### Chemotherapy

**Figure 1:**
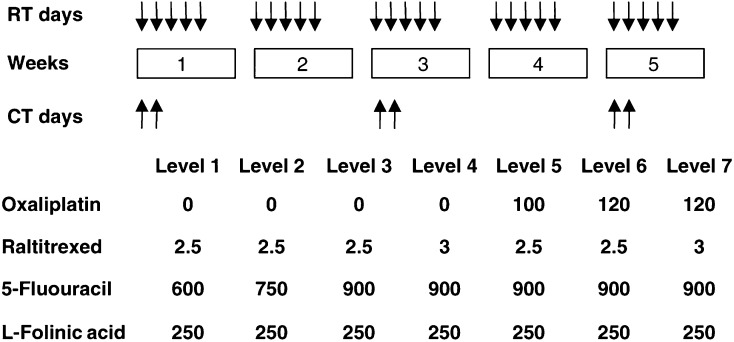
Treatment schedule and design of the dose finding study (doses in mg m^−2^).

Chemotherapy consisted of three biweekly cycles delivered immediately before RT on days 1 and 2 of the first, third and fifth week of RT. In the first four dose levels, patients received only RTX as short intravenous (i.v.) infusion on day 1 and levo-FA (2-h i.v. infusion) followed by FU i.v. bolus on day 2, 24 h after RTX (Figure 1). In the next dose levels, OXA (2-h i.v. infusion) was delivered before RTX on day 1. In case of persistent grade ⩾2 toxicity, according to the National Cancer Institute common toxicity criteria (NCI CTC-Version 2) at the time of recycling, chemotherapy was delayed for 1–2 weeks. Otherwise, chemotherapy was permanently discontinued. A 25% FU dose reduction was applied in subsequent cycles in case of grade 4 neutropenia or anaemia, grade ⩾3 febrile neutropenia or thrombocytopenia, grade ⩾2 cardiac or renal toxicity or grade ⩾3 other non-haematologic toxicities (except for alopecia). At the second appearance of these side effects or after the first appearance of grade ⩾3 sensory neuropathy, a 25% OXA dose reduction was also planned. Doses of RTX and levo-FA were never reduced.

### Surgery

Patients underwent TME 8 weeks after the completion of chemoradiation. An anterior resection (AR) or an APR was performed on the basis of restaging. Intraoperative frozen sections were obtained to assess the resection margins in the case of conservative treatment. Anastomoses were protected by a loop ileostomy, and ileostomy reversal was performed after endoscopic assessment of anastomotic integrity.

### Pathology

Pathologic examination provided a macroscopic description of the mesorectum and of the former tumour-bearing area. For residual tumour, at least four paraffin blocks were processed, and an additional large area block was embedded. If no tumour was visible, the entire suspicious area was sliced and embedded. Circumferential resection margin was examined by sampling a 1 mm thick slice, and lymph nodes were searched by manual dissection (Andreola *et al*, 2001a, 2001b). All resection specimens were examined by the same experienced pathologist, according to a standardised protocol based on tumour node metastasis categories, reporting the number of examined and involved lymph nodes (including the apical node one), the CRM evaluation and the tumour regression grading (TRG). The pathologic records were reported on a standard form for all patients.

Pathologic response was classified as follows: TRG1, complete response with absence of residual cancer cells and fibrosis extending through the wall (regardless of the presence of acellular mucine lakes); TRG2, presence of few residual cancer cells scattered through the fibrosis; TRG3, clear evidence of residual cancer cells, but with predominant fibrosis; TRG4, residual cancer cells outgrowing fibrosis; TRG5, absence of regressive changes (Mandard *et al*, 1994).

### Adjuvant chemotherapy

Four months of adjuvant chemotherapy with weekly FU 370 mg m^−2^ and levo-FA 20 mg m^−2^ (QUASAR Collaborative Group, 2000) were planned only for patients with cT4 lesions, or with pN+ and/or pCRM ⩽1 mm.

### Follow-up

Patients were clinically assessed every 3 months for the first 2 years, every 6 months for the next 2 years and annually thereafter, with pelvic MRI, chest and abdominal CT, CEA serum level and proctoscopy.

### Study design

#### Phase I

Based on pharmacokinetic interactions, escalation of RTX and FU was planned only up to 3 and 900 mg m^−2^, respectively (Schwartz *et al*, 2004). Subsequently, OXA was added and escalated (Figure 1). Consecutive cohorts of three patients were treated at each dose level. If a dose-limiting toxicity (DLT) was observed, this cohort was expanded to six patients. If ⩽2 patients out of six patients experienced a DLT at a given dose level, escalation proceeded. Maximum tolerated dose (MTD) was defined as the dose level at which DLTs occurred in more than one-third of patients. Recommended dose (RD) for the subsequent phase II study was defined as the dose level preceding the MTD. Dose-limiting toxicities were defined as grade 4 neutropenia or anaemia; grade ⩾3 febrile neutropenia or thrombocytopenia; grade ⩾2 renal and cardiac toxicity; grade ⩾3 other non-haematologic toxicity (except for alopecia); delay of more than 2 weeks in chemotherapy recycling; and any other severe adverse events.

#### Phase II

We have chosen as primary end point the achievement of a complete or nearly complete pathologic response, because it has been repeatedly reported to predict the long-term survival of patients (Bouzourene *et al*, 2002; Rödel *et al*, 2005; Vecchio *et al*, 2005). To define the sample size, a Simon's two-stage design was utilised (Simon, 1989), setting *α* and *β* errors as 0.05 and 0.20, and defining as minimum activity of interest (p0) a TRG1–2 rate=30%. To accept the alternative hypothesis (p1) of a TRG1–2 rate ⩾50%, at least six TRG1 or 2 in the first 15 patients, and at least 19 TRG1 or 2 in a total of 46 patients had to be reported. Secondary end points were safety, progression-free survival and OS.

## RESULTS

### Patient characteristics

Between December 2000 and August 2004, 20 patients were enrolled in the phase I study and 31 patients were enrolled in the phase II study (Table 1). In the phase II study, 27 patients were at moderately high or high risk of recurrence according to Gunderson *et al* (2004). Tumour distance from the mesorectal fascia was ⩽5 mm in 19 patients, and from the anal verge was ⩽5 cm in 15 patients.

### Dose escalation and DLTs

In the first four dose levels, no DLT occurred. With the addition of OXA, one of three patients showed grade 4 neutropenia after the second cycle of chemotherapy. Therefore, three more patients were treated with this dose level. Of these last patients, a 65-year-old women suffered from grade 3 diarrhoea, which eventually recovered allowing for treatment completion. At the sixth dose level, two of two patients experienced a DLT: a 77-year-old man had a delay of more than 2 weeks after the first cycle, caused by grade 3 neutropenia, and a 58-year-old man presented grade 3 colitis after the third cycle. Consequently, the previous dose level was considered the RDs (Table 2).

### Toxicity of the phase II study

Acute toxicities are listed in Table 3. Stomatitis, diarrhoea and neutropenia were the only grade 3 or 4 toxicities. Grade 3 stomatitis occurred in one (3%) patient, whereas six (19%) patients experienced grade 3 diarrhoea. Neutropenia was of grade 3 in five (16%) patients and of grade 4 in seven (23%) patients (febrile in two patients). Only one patient required hospitalisation for diarrhoea and concomitant grade 4 febrile neutropenia, and he did not complete RT (cumulative dose, 41.5 Gy). In all other cases, toxicities were easily managed, and resolved in 2–4 days. Grade 1 peripheral neuropathy occurred in five (16%) patients. Eight of 93 cycles of chemotherapy were omitted for toxicity (*n*=6) or refusal (*n*=2), with an RT and chemotherapy compliance of 97 and 91%, respectively. A 25% FU dose reduction was needed in 11 patients.

### Surgical morbidity

There were no intra- or postoperative deaths. Thirteen (37%) patients treated with the RDs experienced complications, including anastomotic fistula (*n*=5), urinary fistula (*n*=1), recto-vaginal fistula (*n*=1), wound dehiscence (*n*=1), presacral abscess (*n*=1), minimal anastomotic stenosis (*n*=3) and anastomotic leakage (in a male patient who refused loop ileostomy). Only one patient required additional surgery.

### Activity

#### Phase I

All 20 patients underwent R0 resection, but liver metastases were diagnosed intraoperatively in two patients. A TRG1 was obtained in six (30%) patients and a TRG2 in two (10%) patients.

#### Phase II

All patients had a TME with complete mesorectum, and the median number of retrieved nodes was 29 (range 10–80). Twenty-nine (93%) patients had an R0 resection, because CRM was ⩽1 mm in two patients. Anterior resection was performed in 25 patients, and a sphincter-preserving procedure could be performed in nine of 15 (60%) patients with tumour located ⩽5 cm from the anal verge. In two patients, APR was mandatory because of baseline sphincter infiltration. Twenty-five patients (81%) obtained a T downstaging (four of six cT4, 19 of 23 cT3 and two of two cT2). Nodal downstaging was detected in 23 of 28 (82%) patients. Positive lymph nodes were detected in only five (16%) patients, one of whom with a single micrometastatic focus. No patient showed an increase of T or N stage. Pathologic evaluation showed a TRG1 in 13 (42%) patients and a TRG2 in nine (29%) patients. Therefore, a TRG1 or 2 was reported in 71% (95% confidence limits, 52–86%) of patients, with no significant correlation with baseline clinical characteristics (Table 4).

### Follow-up

As of November 2005, after a median follow-up of 53 (range 41–59) months, four patients recruited in the phase I study had a distant recurrence (only one had received OXA) and four patients had died (two patients for cancer-unrelated causes). All 31 patients of the phase II study are alive and recurrence-free after a median follow-up of 29 (range 16–41) months.

## DISCUSSION

The results of a recent randomised trial, combining FU with local RT, have further supported preoperative *vs* postoperative treatment of LARC, but have also raised several issues (Sauer *et al*, 2004). In particular, the lack of reduction of distant metastases in this trial strongly supports the exploitation of more active cytotoxic regimens. Moreover, the risk of overestimating the tumour's degree of penetration with EUS points to the need for a more accurate staging of patients. Furthermore, retrospective data have suggested that a combined modality therapy could represent an overtreatment in patients at low risk of local failure after TME alone, such as some tumours staged as T3N0 (Willett *et al*, 1999). In this setting, MRI plays a key role, because it can predict the CRM involvement (Beets-Tan *et al*, 2000), so defining the patients at higher risk of local recurrence when treated with TME plus RT (Marijnen *et al*, 2003), although it has been demonstrated less sensitive and specific at identifying nodal disease and vascular invasion (Brown *et al*, 2003; Branagan *et al*, 2004).

Our phase I–II study was carried out in patients with LARC at high risk of recurrence, as identified by both EUS and MRI. The aim of the phase I study was to determine the MTDs of the combination of RTX, FU/FA and OXA combined with pelvic RT. Raltitrexed and FU were escalated only up to 3 and 900 mg m^−2^, respectively, because a pharmacokinetic analysis has demonstrated that pre-administration of RTX (⩾2.5 mg m^−2^) 24 h before FU (900 mg m^−2^) significantly increased its *C*_max_ and AUC (Schwartz *et al*, 2004).

A grade 3 neutropenia was the only severe toxicity that occurred in the first four dose levels. The safety of the administration of RTX plus FU during RT could likely be explained by the concurrent delivery of FA. Indeed, preclinical studies have shown that the administration of FA 24 h after RTX not only reduced its toxicity (Farrugia *et al*, 2000) but also increased the synergism between RTX and FU (Longo *et al*, 1998). When OXA was added to the combination, the RDs for the phase II study were OXA 100 mg  m^−2^, RTX 2.5 mg m^−2^, FU 900 mg m^−2^ and LFA 250 mg m^−2^, administered every 2 weeks. Notably, these dosages are similar or even greater than those usually utilised in the treatment of metastatic colorectal cancer patients without RT (Cascinu *et al*, 2002; Seitz *et al*, 2002; Goldberg *et al*, 2004; Comella *et al*, 2005).

Neutropenia and diarrhoea were the most frequent grade 3 or 4 adverse events occurring in the phase II study. However, we would underline that in our experience, the occurrence of grade ⩾3 neutropenia was similar to that reported in the MOSAIC trial with FOLFOX4 regimen (André *et al*, 2004), and severe diarrhoea was not higher than that seen in the CAO/AIO/ARO-94 trial with preoperative RT plus FU (Sauer *et al*, 2004). Moreover, in all but one case, toxicity resolved in 2–4 days without hospitalisation. Neurotoxicity was negligible and scored as grade 1 only. The favourable toxicity profile of this combined treatment was reflected by its high compliance and by the acceptable surgical morbidity, with only one patient requiring re-operation.

In the phase II study, we have analysed the pathologic response using the TRG score, because it has been shown to be more accurate in defining the tumour regression after primary therapy, and to predict the long-term outcome (Bouzourene *et al*, 2002; Rödel *et al*, 2005; Vecchio *et al*, 2005). Notably, the number of TRG1 or 2 required by our statistical design had already been reached in the first 31 treated patients, with an overall activity (71%) by far greater than that hypothesised. In addition, such activity has never been reported with preoperative combination chemotherapy and RT (Gerard *et al*, 2003; Rodel *et al*, 2003; Mehta *et al*, 2003; Gambacorta *et al*, 2004a; Aschele *et al*, 2005; Sebag-Montefiore *et al*, 2005). The 95% confidence limits of these results (52–86%) strongly support the conclusion that this approach could be effective in at least half of treated patients. Moreover, it should be stressed that the complete or nearly complete pathologic response rate was obtained in high-risk patients (Phang *et al*, 2002; Gunderson *et al*, 2004). Interestingly, the achievement of TRG1–2 was independent of baseline characteristics. The high activity of this treatment was reflected by the per cent of R0 resection (93%), as well as tumour and nodal downstaging (81 and 82%, respectively). Moreover, 84% of patients showed no nodal involvement, no T or N upstaging was observed, and sphincter preservation was obtained in 60% of patients with low-lying tumours.

One could argue whether a similar activity could be obtained without the inclusion of RTX in the combination. Indeed, several recent phase I–II trials have explored the activity of a combination of OXA and fluoropyrimidines (i.v. FU or oral capecitabine) during preoperative pelvic RT (Gerard *et al*, 2003; Rodel *et al*, 2003; Aschele *et al*, 2005; Sebag-Montefiore *et al*, 2005). Although all these trials have demonstrated the feasibility of such combinations, we would underline that the reported pCR rates (ranging between 7 and 28%) were clearly lower than that achieved in our study. Notably, in all these studies, the OXA dose intensity during radiotherapy ranged from 43 to 60 mg m^−2^ per week, which is similar to that we were able to deliver with our three-drug combination.

On the other hand, a phase II study integrating three cycles of OXA and TOM (without FU/FA) with pelvic RT has reported a pCR in 9 out of 30 (30%) LARC patients (Gambacorta *et al*, 2004a). However, it should be noted that only patients with a limited disease extent (not including T4) were treated in this study. On the contrary, our treatment yielded a 42% TRG1 in an unfavourable patient population.

Furthermore, we would point out the accurate pathologic evaluation we performed, which allows a critical assessment of the primary treatment, and may facilitate interstudy comparison. Indeed, an accurate pathologic analysis allows one to assess the CRM involvement, the number of examined and involved lymph nodes and the quality of surgical performance, providing valuable data that have an impact on outcome (Tepper *et al*, 2001; Nagtegaal *et al*, 2002; Mawdsley *et al*, 2005). It is important to stress that in our study, the resection of mesorectum was complete in all patients, CRM was ⩽1 mm in only two patients and a median number of 29 nodes was retrieved.

Finally, it is remarkable that after a median follow-up of 29 months, all patients of the phase II study are alive and recurrence-free. Of note, only 11 of these patients have received post-resection adjuvant FU/FA.

In conclusion, this study demonstrates that the combination of OXA, RTX and FU/FA with pelvic RT in LARC patients has an acceptable toxicity and an excellent treatment compliance. Moreover, it provides evidence of high clinical activity in patients selected for high risk of recurrence. On these bases, we have decided to complete patient accrual up to the planned sample size, testing a slight dose reduction of FU (800 mg m^−2^) to further improve the safety of this combined treatment.

## Figures and Tables

**Table 1:** Characteristics of patients entered in the phase I and II studies

**Characteristics**	**Phase I, no. (%)**	**Phase II, no. (%)**
Total patients	20 (100)	31 (100)
Males	14 (70)	16 (52)
Females	6 (30)	15 (48)
Median age (years) (range)	63 (41–77)	56 (29–74)
		
*ECOG performance status*		
0	9 (45)	12 (39)
1	11 (55)	17 (55)
2	0	2 (6)
		
*Risk of failure*^a^ *and TNM clinical staging*		
Low risk		
T1–2/N0	1 (5)	0
		
Intermediate risk		
T1–2/N1	0	1 (3)
T3N0	8	3 (10)
		
Moderately high risk		
T1–2/N2	0	0
T3N1	7 (35)	14 (45)
T4N0	3 (15)	0
		
High risk		
T3N2	0	7 (23)
T4N1–2	1 (5)	6 (19)
		
*Distance of primary tumour from anal verge*		
⩽5 cm	11 (55)	15 (48)
6–10 cm	8 (40)	12 (39)
>10 cm	1 (5)	4 (13)
		
*Distance of tumour from mesorectal fascia* b		
⩽5 cm	3 (15)	19 (61)
>5 cm	3 (15)	9 (29)
Not evaluated	14 (70)	3 (10)
		
*CEA*		
=5 UI	11 (55)	21 (68)
>5 UI	9 (45)	10 (32)

CEA=carcinoembryonic antigen assay; ECOG=Eastern Cooperative Oncology Group; TNM=tumour node metastasis.

a Risk of failure evaluated according to Gunderson's classification.

b Evaluated by MRI (only in patient treated with oxaliplatin). In two patients treated in the phase I and in three patients in the phase II studies, MRI was not performed because of metal prosthesis in four and metal stitches in the other.

**Table 2:** Toxicity by dose levels

	**NCI-CTC grade**								
	**Dose levels 1–4**			**Dose level 5**			**Dose level 6**		
	**(*n*=12)**			**(*n*=6)**			**(*n*=2)**		
**Toxicity**	**Grade 1/2**	**Grade 3**	**Grade 4**	**Grade 1/2**	**Grade 3**	**Grade 4**	**Grade 1/2**	**Grade 3**	**Grade 4**
Haematologic									
Neutropenia	2	1			2	1		2	
Febrile neutropenia				1					
Thrombocytopenia	1			1			1		
Gastrointestinal									
Nausea/vomiting	7			2					
Colitis				2				1	
Diarrhoea	1			3	1		1		
Stomatitis	1			1					
Proctitis	1			1					
Genitourinary									
Cystitis				1					
Skin, local toxicity	1								
Fatigue/asthenia							1		
Fever	1								

NCI-CTC=National Cancer Institute Common Toxicity Criteria.

*Note*: Most severe toxicity of each type experienced by each patient.

**Table 3:** Worst toxicities by patients reported in the phase II study

	**NCI-CTC grade**			
	**Grade 1**	**Grade 2**	**Grade 3**	**Grade 4**
**Toxicity**	**no. (%)**	**no. (%)**	**no. (%)**	**no. (%)**
Haematologic				
Neutropenia	4 (13)	1 (3)	5 (16)	5 (16)
Febrile neutropenia	1 (3)	2 (6)		2 (6)
Thrombocytopenia	2 (6)	3 (10)		
Gastrointestinal				
Nausea/vomiting	11 (35)	4 (13)		
Colitis	1 (3)	2 (6)		
Diarrhoea	2 (6)	5 (16)	6 (19)	
Stomatitis			1 (3)	
Proctitis	3 (10)	2 (6)		
Skin, local toxicity	1 (3)	2 (6)		
Fatigue/asthenia	3 (10)	1 (3)		
Fever	1 (3)	2 (6)		
Paresthesia/dysesthesia	5 (16)			

NCI-CTC=National Cancer Institute Common Toxicity Criteria.

**Table 4:** Correlation between pretreatment clinical characteristics and pathological findings in the phase II study (*n*=31)

	**TRG1–2**	**TRG3–4**
**Clinical characteristics (number of patients)**	**no. (%)**	**no. (%)**
cTNM staging – Gunderson risk of failure		
T1–2/N1; T3N0 – intermediate	3 (75)	1 (25)
T1–2/N2; T3N1; T4N0 – moderately high (14)	11 (79)	3 (21)
T3N2; T4N1–2 – high (13)	8 (61)	5 (39)
		
Distance of primary tumour from anal verge		
=5 cm (14)	11 (79)	3 (21)
>5 cm (17)	11 (65)	6 (35)
		
Distance of tumour from mesorectal fascia^a^		
=5 mm (19)	15 (79)	4 (21)
>5 mm (9)	6 (67)	3 (33)

TNM=tumour node metastasis.

a MRI was not performed in three patients, for the presence of metal prosthesis (two patients) and metal stitches (one patient).
